# Selection and characterization of llama single domain antibodies against N-terminal huntingtin

**DOI:** 10.1007/s10072-014-1971-6

**Published:** 2014-10-08

**Authors:** Menno H. Schut, Barry A. Pepers, Rinse Klooster, Silvère M. van der Maarel, Mohamed el Khatabi, Theo Verrips, Johan T. den Dunnen, Gert-Jan B. van Ommen, Willeke M. C. van Roon-Mom

**Affiliations:** 1 Department of Human Genetics, Center for Human and Clinical Genetics, Leiden University Medical Center, Albinusdreef 2, 2333 ZA Leiden, The Netherlands; 2 Leiden Genome Technology Center, Center for Human and Clinical Genetics, Leiden University Medical Center, Albinusdreef 2, 2333 ZA Leiden, The Netherlands; 3 QVQ BV, Padualaan 8, 3584 CH Utrecht, The Netherlands; 4 Merus BV, Padualaan 8, 3584 CH Utrecht, The Netherlands

**Keywords:** VHH, Huntington disease, PolyQ, N-terminal huntingtin, Huntingtin

## Abstract

Huntington disease is caused by expansion of a CAG repeat in the *huntingtin* gene that is translated into an elongated polyglutamine stretch within the N-terminal domain of the huntingtin protein. The mutation is thought to introduce a gain-of-toxic function in the mutant huntingtin protein, and blocking this toxicity by antibody binding could alleviate Huntington disease pathology. Llama single domain antibodies (VHH) directed against mutant huntingtin are interesting candidates as therapeutic agents or research tools in Huntington disease because of their small size, high thermostability, low cost of production, possibility of intracellular expression, and potency of blood-brain barrier passage. We have selected VHH from llama phage display libraries that specifically target the N-terminal domain of the huntingtin protein. Our VHH are capable of binding wild-type and mutant human huntingtin under native and denatured conditions and can be used in Huntington disease studies as a novel antibody that is easy to produce and manipulate.

**Electronic supplementary material:**

The online version of this article (doi:10.1007/s10072-014-1971-6) contains supplementary material, which is available to authorized users.

## Introduction

Huntington disease (HD) is caused by expansion of a CAG repeat within the first exon of the *huntingtin* gene (4p16.3) [1]. This mutation results in an expanded polyglutamine repeat (polyQ) at the N-terminus of the huntingtin protein (htt), causing HD pathology through a toxic gain-of-function mechanism [2]. Antibody binding could reduce toxicity of the mutant htt protein. Messer et al. showed that a single chain Fv antibody construct, selected against the first 17 N-terminal htt amino acids was capable of reducing HD pathogenesis in various HD models [3, 4]. In our study we make use of llama single domain antibody fragments called VHH [5]. VHH contain four framework regions (FR1–4) for structural integrity and three variable complement determining regions (CDR1–3) that usually determine epitope binding. VHH have distinctive advantages compared with other antibody classes. VHH are thermostable, only ~16 kD in size and their single domain nature simplifies selection and production [6, 7]. VHH have been used for diseases such as oculopharyngeal muscular dystrophy (OPMD), which shares characteristics with HD. OPMD is caused by expansion of a triplet repeat in the *PABPN1* gene that encodes for a polyalanine repeat at the N-terminus of the polyA binding nuclear 1-protein (PABN1). VHH binding to an α-helical domain of mutant PABN1 prevented aggregation [8], and alleviated OPMD pathology in a *droso* *philia* model [9]. In the current study, we have selected VHH against the N-terminal domain of htt from llama phage display libraries. We show that high resolution melting curve analysis (HRMCA) [10] can successfully identify identical clones prior to sequencing. Our VHH can bind both endogenous and purified human wild-type and mutant htt at an epitope located between amino acids 49–148 and can co- immunoprecipitate htt from human HD brain lysates.

## Results

### Selection of VHH against N-terminal huntingtin

**Fig. 1:**
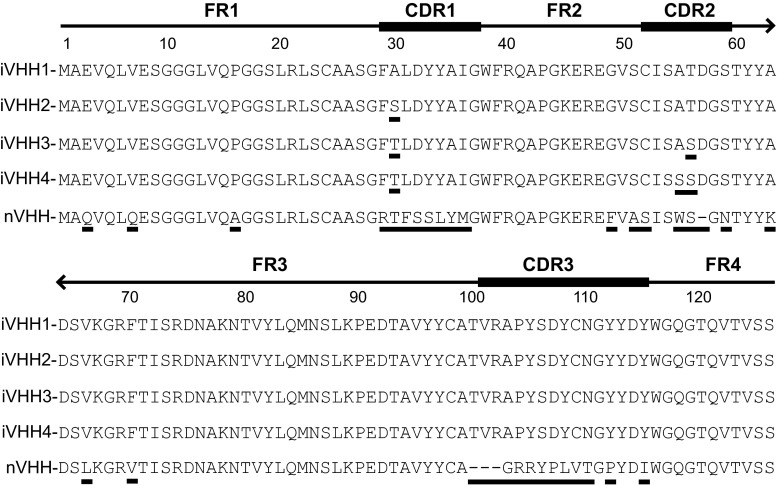
VHH protein sequences. iVHH 1–4 were selected from an immunized llama phage display library. nVHH was selected from a non-immunized llama phage display library. *Underscored* amino acid position differs from iVHH1. Amino acid positions, framework (FR, *thin line*) and complementary determining regions (CDR, *thick line*) are according to Kabat [25]. −, deletion

The phage-VHH (P-VHH) display library originated from llamas pre-immunized with an *Escherichia* *coli* produced N-terminal htt protein fragment consisting of the first 548 amino acids with 46 polyQs. We performed four different selections; each selection involved two rounds using either a wild-type or mutant N-terminal htt fragment. Enrichment of P-VHH after two rounds of selection was similar for direct coating or pre-capturing of N-terminal htt in the first round, with the optimal concentration being 5 µg N-terminal htt (Online Resource 1a). P-VHH output numbers of up to 3 × 10^4^ were obtained. Screening ELISA revealed that on average, 20 % of selected clones bound htt (Online Resource 1b). Further screening by HRMCA (Online Resource 1c), followed by sequence analysis, revealed four htt specific VHH, (immune)VHH1-4. These differed by one, two, or three amino acid substitutions in the CDR1 and CDR2, while the CDR3 was identical (Fig. 1). The negative control VHH (n-VHH) was selected previously from a naive llama phage display library [10].

### Specificity of monoclonal iVHH for N-terminal huntingtin

**Fig. 2:**
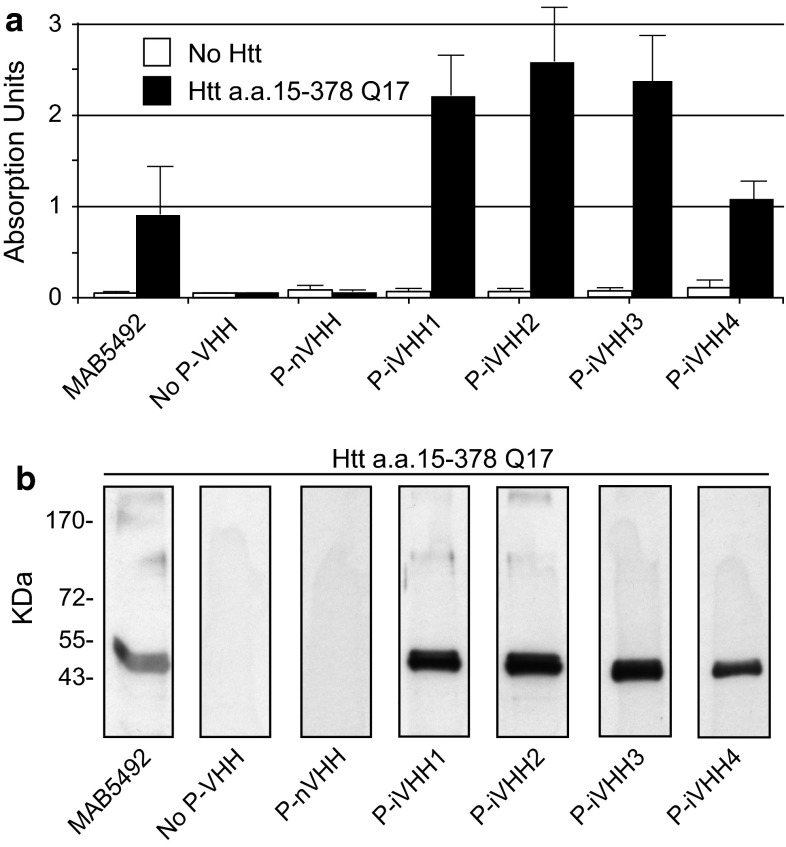
VHH specificity for N-terminal htt. Assays were performed on a recombinant N-terminal htt fragment consisting of amino acids 15–378 with a polyQ length of 17 (htt a.a. 15–378 Q17). Positive control: MAB5492. Negative control: No P-VHH or P-nVHH. **a** ELISA on wells with (*black bars*), or without (*white bars*) N-terminal htt. *Bars* represent mean ELISA signal from two independent ELISA assays with standard deviation. Each assay was performed in triplicate. ELISA absorption units are measured at *λ* = 490 nm. **b** Western blotting on N-terminal htt. Blots were performed twice. *kDa* Molecular weight (kilodalton)

To investigate if the iVHH specifically bind htt, we performed ELISA and western blot analysis on a normal and mutant N-terminal htt fragment using P-iVHH [11]. ELISA resulted in a positive signal for all P-iVHH (Fig. 2a, Online Resource 2a). P-iVHH1, 2 and 3 showed an equally strong ELISA signal, whereas P-iVHH4 gave a weaker signal. On western blot, all P-iVHH showed a band that matched the band obtained with the known htt antibody MAB5492 (Fig. 2b, Online Resource 2b). Western blotting results were in agreement with ELISA results.

### Epitope determination of iVHH on N-terminal huntingtin

**Fig. 3:**
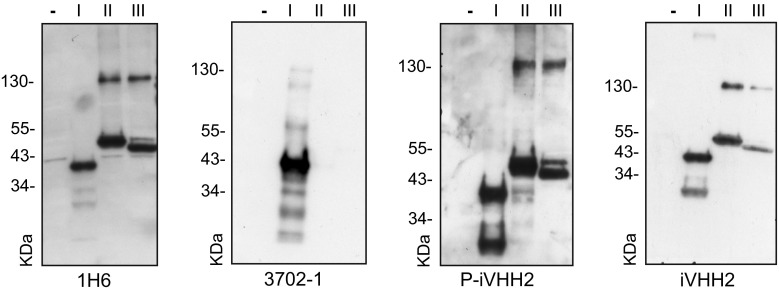
VHH epitope determination. Western blots performed on no htt (−), htt a.a. 1–148 Q46 (I), htt a.a. 15–378 Q17 (II), and htt a.a. 49–415 (III). Primary antibody indicated below each blot. Blots were performed twice. *kDa* Molecular weight (kilodalton)

To determine the epitope of our iVHH, we performed western blotting using three different N-terminal htt fragments. Each fragment had a partially overlapping sequence with the next fragment (Fig. 3). All fragments were recognized by the 1H6 antibody [12] while 3702–1, that binds htt at the N-terminal 13 amino acids (Online Resource 3a), only recognized fragment I. All P-iVHH recognized all fragments (Fig. 3, Online Resource 3b) indicating that their epitope is located within the overlapping region of fragments I, II and III that consists of amino acids 49–148. Finally, western blotting with monomeric iVHH was performed. VHH were produced with an average concentration of 1.1 µg/µl. Western blotting using iVHH instead of P-iVHH recognized htt fragments I, II, and III but with less back ground staining.

### iVHH detection of endogenous huntingtin in human HD brain lysates

**Fig. 4:**
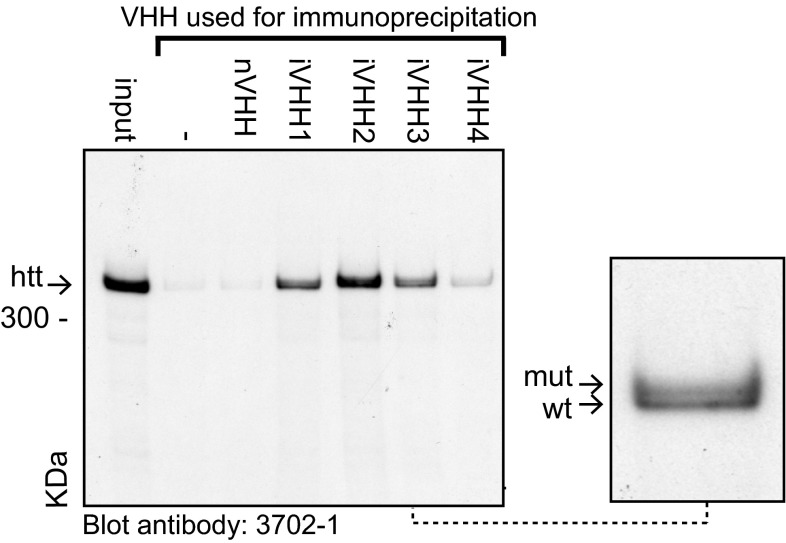
Immunoprecipitation of endogenous human htt with VHH. Western blot analysis of VHH-htt immunoprecipitation complexes. VHH used for htt immunoprecipitation indicated at *top*, Input, 10 µg of brain lysate; –, No VHH. *Arrow* indicates full-length htt. *kDa* Molecular weight (kilodalton). Blot was analysed with 3702-1 anti htt antibody. *Right bracket* 3× enlargement of the iVHH3 immunoprecipitation result, showing wild-type (wt) and mutant (mut) huntingtin

Next, we analysed if our iVHH were capable of binding endogenous human htt. We performed immunoprecipitation experiments using monomeric iVHH and post-mortem human HD brain lysates. Non-specific binding of htt to the protA-beads without VHH (Fig. 4, –lane) was low. Immunoprecipitation in the presence of iVHH resulted in wild-type and mutant full-length htt band on western blot, while the negative control VHH (nVHH) did not show a full-length htt band (Fig. 4, Online Resource 4). This shows that, in concordance with previous ELISA and western blot results on N-terminal wild-type and mutant htt fragments, our iVHH recognize both wild-type and mutant endogenous full-length htt. Additionally, immunoprecipitation with iVHH4 was less efficient compared with the other iVHH.

## Discussion

To our knowledge, our study is the first in which VHH have been selected against N-terminal htt protein fragments from an immunized llama phage display library. Monoclonal antibodies are increasingly used as therapeutic agents [13]. Proof of concept for using antibodies in HD has been provided [3, 4]. Whilst this is promising, there are high demands on any therapeutic or imaging antibody. Because HD is a brain disorder, it is important to cross the blood–brain barrier (BBB). Some VHH were able to cross the BBB [14, 15]. Also, VHH diffusion throughout the brain, and cellular uptake were demonstrated [15]. Although VHH selection from non-immune phage-VHH libraries is possible [16], selection from immune llama phage-VHH libraries is expected to yield more high-affinity binders because of affinity maturation. However, diversity will be less as affinity maturation selects for the strongest binder. This is reflected in our results. Our iVHH share a high degree of sequence homology. This indicates that the binding epitope of our iVHH is very similar. Our iVHH bind full-length and N-terminal htt in a native and denatured conformation. Binding characteristics for iVHH1-3 were comparable, whereas iVHH4 showed a lower binding efficiency. This is probably due to a single amino acid change in iVHH4 at position 55 in CDR2 where the a-polar alanine was replaced with a polar serine. Because both alanine and serine have side-chains of comparable sizes, it is conceivable that the lower binding efficiency of iVHH4 is due to a shift from an a-polar to polar amino acid. The epitope of all iVHH was mapped to a region between amino acids 49 and 148 downstream of the polyQ repeat, explaining why our iVHH bind both wild-type and mutant htt. In this region, htt contains several domains associated with HD pathogenesis. There is a proline rich region involved in sequestration of other proteins into htt aggregates [17]. Furthermore, proteolytic cleavage between amino acids 104 and 114 was linked with formation of intranuclear aggregates associated with increased toxicity [12]. Binding of our iVHH in this region could alleviate HD pathogenesis. However, it has to be noted that our huntingtin-specific VHH could also block neuroprotective properties of wild-type htt [18]. Additional research is needed to assess the therapeutic properties of our iVHH or their possible applications in HD research. We have demonstrated the feasibility of using VHH for immunoprecipitation, eliminating interference by IgG bands in the subsequent western blot [19]. Furthermore, since VHH have been used as imaging agent to stain amyloid-β deposits in vivo in an Alzheimer disease mouse model [20], our iVHH could be an interesting in vivo imaging tool in HD models to visualize the htt protein.

## Materials and methods

### N-terminal htt fragments

A *HTT* reference sequence with 23 polyQs was used. For PCR primers see Table 1. PCR products were ligated directly into the pGEM-T easy vector (Promega, Madison, WI, USA), digested with *Nco*I and *Sal*I (Fermentas, St. Leon-Rot, Germany), and ligated into a *Nco*I/*Xho*I (Fermentas) pre-digested pIVEX 1.3 vector. Clones were confirmed by sequence analysis. Htt protein fragments were produced with the RTS-100 wheat germ CECF kit (5 PRIME, Gaitersburg, MD, USA) using the ProteoMaster rapid translation system (Roche). To produce the N-terminal htt 1–148 Q46 protein fragment, the HD1955 pCI construct consisting of *HTT* nucleotides 1–1640 [21] was cloned into a pRP261 vector using the *Nco*I/*Sal*I restriction sites, and re-cloned into the pET28 vector using the *Bam*HI/*Sal*I restriction sites. The HD1955-pET28 construct was digested with *Xho*I and self-religated, resulting in the HD828-pET28 construct. Production and purification were performed in BL21 codon+ *E.coli* cells as described for VHH. To prevent aggregation, dialysis was performed in PBS + 0.5 % Sarkosyl (Sigma–Aldrich).

**Table 1:** Construction of N-terminal Htt fragments

N-terminal htt fragment	Forward primer	Reverse primer
a.a. 1–318 (Q17/Q43)	TATGGCGACCCTGGAAA	GTCGACGAGCAGCACGCCAAGA
a.a. 15–378 (Q17/Q43)	CAAGTCCTTCCAGCAGCA	GTCGACGGCTCCGGTCACAACA
a.a. 49–415 (no polyQ)	GCCGCCTCCTCAGCTTC	GTCGACGCCACCAGACTCCTCCTT

*a*.*a*. amino acid, *Q17/Q43* polyQ stretch, *Underscored* *Sal*I-site

### VHH selection

Selections were performed as described [16]. The first selection round involved a direct coating of NUNC maxisorp plates (Thermo fisher Scientific, Rochester, NY, USA) with 10, 5, or 2.5 µg of antigen, or a pre-capturing of 10 µg antigen with 2, 1, or 0.5 µg of coated 1H6 antibody (Abnova, Taipei, Taiwan). Phage-VHH (P-VHH) from the first round were produced and purified as described [22] and subjected to a second round of selection with 5, 2.5, or 1 µg of directly-coated antigen. Purified P-VHH were stored at −20 °C in PBS containing 10 % glycerol. TG1 *E. coli* cells were infected with phage-VHH from the second selection round and plated on LB/Agar containing ampicillin. Ninety-four randomly selected clones were tested as described [10]. As secondary antibody for the screening ELISA, we used HRP conjugated mouse anti M13 (GE Healthcare, Buckinghamshire, UK). *VHH* DNA was purified using the Nucleospin Plasmid purification kit (Macherey–Nagel, Duren, Germany) according to manufacturer’s instructions. DNA was sequenced using primer M13REV (CAGGAAACAGCTATGAC). DNA to protein conversion: http://www.bioinformatics.picr.man.ac.uk/research/software/tools/sequenceconverter.html. VHH sequence alignment: http://www.ebi.ac.uk/Tools/msa/clustalW2.

### VHH production

PCR was performed on M13 plasmid using primers iVHH-FW (CGGAATTCCTTTAGTTGTTCCA), and iVHH-Rev (CACATCATCATCACCATCACG), or nVHH-FW (CGCTGGATTGTTATTACTCGC) and nVHH-Rev (CCTCAGAACCCAAGACCA). PCR fragments were cloned into pUR5850 [23] by *Sfi*I and *Bst*EII (Fermentas) digestion and ligation. Clones were verified by sequence analysis and transferred into Neb5 *E. coli* for production. VHH were purified using the His6-tag with TALON metal affinity resin (Clontech, Mountain View CA, USA) according to the manufacturer’s instructions using 50 mM NaPO4, 0.3 M NaCl, pH7 as wash buffer. Wash buffer with 150 mM imidazole was used as elution buffer. Pooled eluates were dialyzed against PBS using Cellu-Sep dialyze-tube MWCO 3500 (Interchim, MontluÇon, France). VHH production was checked on Coomassie staining (PageBlue, Thermo-Scientific) and western blotting against the VSV-tag. VHH was quantified with bicinchoninic assay (BCA, Thermo-Scientific). VHH were stored in 5 % glycerol at −20 °C.

### ELISA

A 96-well NUNC maxisorp plate was coated with 0.1 µg/well of antigen. Staining was performed using P-VHH diluted 1:20, followed by HRP conjugated mouse anti M13 (Millipore Billerica, MA, USA). ELISA signal was visualized with *o*-Phenylenediamine (OPD, Sigma–Aldrich). Optical density at *λ* = 490 nm was measured using a plate reader (Biotek, Winooski, USA). The huntingtin-specific antibody MAB5492 (Millipore) was used as positive control.

### Western blot

N-terminal htt fragments were run on a 10 % SDS-PAGE gel and proteins were blotted onto nitrocellulose membrane (#170-4159, Bio-Rad, Hercules, CA, USA). Blots were blocked with 4 % non-fat milk (Nutricia, Schiphol, The Netherlands) in TBST. Primary antibodies: 3702-1 (Epitomics, Burlinggame, CA, USA), MAB5492 (Millipore), 1H6 (Abnova, Taipei city, Taiwan), P-VHH or 20 ng/µl VHH. Blots probed with VHH were subsequently incubated with mouse anti VSV (Cell signalling). Secondary antibodies: HRP conjugated goat anti-mouse, goat anti-rabbit (Santa-Cruz), or mouse anti-M13 (Millipore). Bands were detected with Amersham ECL (#RPN 2132, GE healthcare) and Hyperfilm ECL (#28906837, GE healthcare).

### Immunoprecipitation assay

Human post-mortem brain tissue from the middle temporal gyrus of a 67 year old female HD subject (CAG1: 15, CAG2: 42) was obtained with the families full consent and the ethical approval of the various institutional Ethics Committees. Post-mortem delay was 9 h. VHH (15 µg) was bound to 10 µl bed volume of protein A sepharose beads (GE Healthcare), and incubated for 90 min with 100 µg of post-mortem human HD brain tissue lysate in PBS. Western blot analysis of the VHH-htt complexes was performed as described above, where SDS-PAGE was performed according to [24].

## Electronic supplementary material

Below is the link to the electronic supplementary material.

Electronic supplemental figure 1. Selection of P-iVHH by two rounds of panning against wild type N-terminal huntingtin. **a** TG1 *E.coli* cells infected with P-VHH were diluted as indicated and spotted on agar medium selective for presence of P-VHH. Round 1: P-VHH from the llama phage display bank panned against three different concentrations of htt a.a. 15-378 Q17. Most P-VHH are recovered using 5 µg of N-term htt (indicated in red). Inp 10^−6^ / ^−8^ = cells infected with whole phage display bank library diluted 10^6^ or 10^8^ x. Round 2: P-VHH from the most efficient first round selection were enriched and panned for a second time against htt a.a. 15-378 Q17 at indicated concentrations. Most P-VHH are recovered at a htt fragment concentration of 5 µg (indicated in blue). - = cells infected with enriched first round P-VHH selected against a blank well (background). Inp 10^−8^ = cells infected with whole round 1 P-VHH diluted 10^8^ x. **b** Screening ELISA of 94 individual P-VHH clones from round 2. There were 13 (14%) ELISA positive clones (AU490>0.4) detected. **c** High resolution melting curve analysis (HRMCA) of ELISA positive clones revealed two groups of similar clones; blue (7 clones) and red (3 clones), and three unique VHH (green, pink and grey) (EPS 9306 kb)

Electronic supplemental figure 2. Binding of P-VHH to N-terminal Htt fragment with elongated polyQ. Assays were performed on a recombinant N-terminal htt fragment consisting of amino acids 15 to 378 with a polyQ length of 43 (htt a.a. 15-378 Q43). Anti htt antibody MAB5492 served as positive control. Assays performed without P-VHH or the non-binding P-nVHH served as negative control. **a** ELISA with P-VHH on wells with (grey bars), or without (white bars) htt a.a. 15-378 Q43. Bars represent mean ELISA signal from two independent ELISA assays with standard deviation. Each assay was performed in triplicate. ELISA absorption units are measured at λ=490nm **b** Western blotting with P-VHH on htt a.a. 15-378 Q43. All blots were performed twice. kDa = running height in kilodalton (EPS 4686 kb)

Electronic supplemental figure 3. Epitope determination of 3702-1 and VHH antibodies. **a** Western blot on five different N-terminal htt fragments: htt a.a. 1 to 318 with wild type (Q17) and mutant (Q43) polyQ, htt a.a. 15 to 378 with wild type (Q17) and mutant (Q43) polyQ and htt a.a. 49-415 without the polyQ. MAB5492 (left bracket) binds all htt fragments. 3702-1 (right bracket) only binds htt a.a. 1 to 318 with either the wild type or mutant polyQ. **b** Epitope determination of P-iVHH1, 3 and 4. Fragments: I = N-terminal htt fragment with a.a. 1 to 148 with a mutant polyQ (Q46). II = N-terminal htt fragment with a.a. 15 to 378 with a wild type polyQ (Q17). III = htt fragment with a.a. 49 to 415 without polyQ stretch. - = no htt fragment. Blot performed with non-binding P-nVHH served as a negative control. All blots were performed twice (EPS 11320 kb)

Electronic supplemental figure 4. Immunoprecipitation of human full length htt with VHH. Input, -, nVHH, iVHH1-4 are shown in figure 4. VHH “X” corresponds to iVHH2 produced from the M13-vector. VHH produced from the M13-vector are less pure compared with VHH produced from pUR5850, hence the band intensity of VHH “X” is lower compared with iVHH2. Because the comparison between different VHH production vectors was outside the scope of this manuscript, we removed VHH X from figure 4 (EPS 4158 kb)
